# Re: “Challenges of addressing neglected tropical diseases amidst the COVID-19 pandemic in Africa: A case of Chagas Disease”

**DOI:** 10.1016/j.amsu.2022.104804

**Published:** 2022-10-31

**Authors:** Alejandro Marcel Hasslocher-Moreno

**Affiliations:** Clinical Research Laboratory of Chagas Disease, Evandro Chagas National Institute of Infectious Diseases, Oswaldo Cruz Foundation, Rio de Janeiro, Brazil

Dear Editor,

We read with interest the recent article of Pradhan et al. [1] “Challenges of addressing neglected tropical diseases amidst the COVID-19 pandemic in Africa: A case of Chagas Disease”. Regarding the article, the authors signal that the COVID-19 pandemic has hampered progress in Chagas disease prevention program and, in addition, to anticipating a reversal in progress achieved in elimination efforts.

First, the authors approach Chagas disease (CD) without considering the different contexts in which the disease is present. Contrary to what the authors claim, that CD would be preferentially in rural areas, the process of CD urbanization in the last decades of the 20th century has increased the number of patients with CD in urban cities. This new urban context has also prompted the modification of the clinical-epidemiological profile of patients with CD, evidenced by changes in work activities, food consumption patterns, increased age, significant prevalence of comorbidities, and social determinants as a whole [2]. Second, the natural history of CD has an acute and chronic phase. The chronic phase occurs after the regression of the acute phase and has four well-defined clinical forms: the indeterminate form, in which the individual does not show symptoms and signs of CD; the cardiac form, which frequently involves rhythm and/or conduction heart disorders, left ventricular systolic dysfunction with or without heart failure, and thromboembolic phenomena; the digestive form, which involves peristalsis dysfunction of the esophagus and or intestine; and the mixed form, when cardiac and digestive manifestations occur simultaneously [3]. Therefore, the authors misinterpret government strategies to combat Chagas disease by mixing vector surveillance actions with patient diagnosis and treatment procedures. Remember that we must not forget that care for acute illness, from a therapeutic point of view, is different from that for chronic illness, as well as between clinical forms with special attention to the cardiac form [4].

Pradhan et al. mention that they did a thorough review of articles published in indexed journals, but they do not indicate which period was studied, which databases were consulted or which keywords were used. Recent studies that addressed the *Trypanosoma cruzi*/covid 19 co-infection were not cited by the authors [[5], [6], [7]]. These studies suggest that the possible cardiovascular involvement in co-infected patients would not lead to a higher risk of hospitalization and increased mortality, as indicated by Pradhan et al.

Finally, the authors emphasize acute co-infection with Trypanosoma cruzi and Covid 19, commenting on the possibility of trypanocidal treatment to increase hepatotoxicity. I consider the probability of this co-infection to be very low, as well as the hepatotoxic effect, which is usually mild and does not cause treatment interruption [8].

## Provenance and peer review

Not commissioned, externally peer reviewed.

## Ethical approval

Not applicable.

## Please state any sources of funding for your research

None.

## Author contribution

Alejandro Marcel Hasslocher-Moreno wrote the Letter to the editor.

## Please state any conflicts of interest

The author declares no conflict of interest.

## Registration of research studies

1. Name of the registry: Not applicable.
2. Unique Identifying number or registration ID: Not applicable.
3. Hyperlink to your specific registration (must be publicly accessible and will be checked): Not applicable.

## Guarantor

This is a Letter to editor and has no associated data.

## Consent

Not applicable.
